# Burnout: a reality among physicians and other health professionals

**DOI:** 10.1192/j.eurpsy.2023.2036

**Published:** 2023-07-19

**Authors:** P. Perestrelo Passos, M. J. Amorim, F. Araújo

**Affiliations:** 1 Unidade Local de Saúde do Alto Minho, Viana do Castelo

## Abstract

**Introduction:**

Burnout is a syndrome that results from chronic stress at work, with several consequences to workers’ well-being and health. It is included in the 11th Revision of the International Classification of Diseases (ICD-11) as an occupational phenomenon and is described in the chapter «Factors influencing health status or contact with health services», which includes reasons for which people contact health services but that are not classed as illnesses or health conditions. Burnout isn’t classified as a medical condition.

**Objectives:**

To assess the consequences of health professionals’ burnout: it’s impact at personal and professional level.

**Methods:**

Non-systematic literature review, available in English, using the PubMed database. Key search terms included burnout; physician; psychiatrist; healthcare; depression; suicide.

**Results:**

Burnout is a syndrome conceptualized as resulting from chronic workplace stress that has not been successfully managed. It is characterized by three dimensions: feelings of energy depletion or exhaustion; increased mental distance from one’s job, or feelings of negativism or cynicism related to one’s job; and reduced professional efficacy. Burnout is particularly common on physicians and in other health professionals, like nurses. This problem represents a public health crisis with negative impacts on individual health professionals, patients and healthcare organizations and systems. Systems factors that play a role in burnout include work compression, demands of electronic health records, production pressure and lack of control over one’s professional life.

**Conclusions:**

Physician burnout is an under-recognized and under-reported problem, and, unfortunately, physicians often do not recognize symptoms of burnout, and even less often do they seek help. Burnout refers specifically to phenomena in the occupational context and should not be applied to describe experiences in other areas of life. There are different clinical forms of burnout and various therapeutic strategies. The individual and social impacts of burnout highlight the need for preventive interventions and early identification of this health condition in the work environment. Psychiatrists play a key role in the multidisciplinary diagnosis and treatment of burnout.

**Disclosure of Interest:**

None Declared

